# Medical Education and State Medical Examinations

**Published:** 1897-09

**Authors:** William Warren Potter

**Affiliations:** Buffalo, N. Y., Member New York State Medical Examining and Licensing Board


### MEDICAL EDUCATION AND STATE MEDICAL EXAMINATIONS.

Conducted by WILLIAM WARREN POTTER, M. D., Buffalo, N. Y.,
Member New York State Medical Examining and Licensing Board.

### RIGHT OF APPEAL FROM MEDICAL BOARD.

DOES the Montana statute allow an appeal to an applicant (*Jour. Am. Med. Assn.*) who has been refused a certificate by the medical board authorising him to practise medicine and surgery in the state on the ground that the applicant's examination papers show that he has not the requisite learning to entitle him to such certificate ? This question was raised in the case of State v. District Court, and was passed upon by the Supreme Court of Montana. Its decision is of increased importance because a number of other states have similar statutes and this appears to be the first authoritative adjudication of the question. The language especially involved is as follows : "In all cases of the refusal or revocation of a certificate to practise medicine by the said board, the person aggrieved thereby may appeal from the decision of the board to the district court of the county in which such revocation or refusal was made." It was argued by the attorney-general that this provision only gave the right of appeal where the certificate was refused or revoked by the board for unprofessional, dishonorable or immoral conduct, and that there was no appeal from the refusal of the board to issue a certificate on the ground of incompetency of the applicant. But, in examining the statute, the Supreme Court said it found no language that restricted the right of appeal to any particular class of cases. The terms of the statute are general, giving the right of appeal "in all cases of the refusal or revocation of a certificate to practise medicine by the said board." So it holds that it gives the right of appeal in the class of cases referred to in the question. Nay more, it holds that the trial in the district court, awkward, difficult and unsatisfactory as same might be, would have to be *de novo.* Whether or not such laws are wise or unwise, politic or impolitic, it says is for the legislature to determine. Justice Hunt adds, in a concurring opinion, that judges often have to pass upon equally difficult questions, involving study and knowledge of abstruse sciences, and when these difficult problems arise they are permitted to call to their aid those who are most proficient and experienced in the branches of knowledge required to settle them. Nor does he think that courts will be disposed to reverse medical boards if they can help doing it, where there is no wilful wrong or prejudice proved.

---

### A NEW SYSTEM OF ENTRANCE EXAMINATION AT THE UNIVERSITY OF PENNSYLVANIA.

BEGINNING with the first term of the session of the University of Pennsylvania for 1899, a new system of entrance requirements is to go into effect in the college, the law school and the medical department. The inauguration of this new plan in these departments will mark an important epoch in the history of higher education, and will place the University of Pennsylvania in the front rank of the leading universities of the world.

The new system is quite a radical departure from the present entrance requirements, but by the time it is to go into effect these tests of a student's education will have been yearly increased until the proper requirements have been secured to justify the application of the new plan.

In order that the new system may go into effect in the college as soon as possible, arrangements have been made by which candidates from any school wishing to enter in 1898 may, on request of that school, be examined according to this new plan. The requirements under this plan are in accord with the scheme adopted by the confidence of colleges held for the purpose of establishing a uniform standard of admission.

Those subjects forming part of the entrance requirements to some or all courses at the university, such as English grammar and reading, solid geometry and plane trigonometry and physics, undergo no change. For the course in science and technology the requirements in Latin grammar, Cesar and Virgil will remain the same as heretofore.

Under the new system the requirements for the college and law school will be as follows : English grammar (as in Abbott's How to Parse, or Murray's Advanced Lessons in English Composition. Analysis and Grammar.)

English literature.—The candidate for 1897 must evince a general knowledge of the following works and their authors : Shakespeare's As You Like It, DeFoe's History of the Plague in London, Irving's Tales of a Traveler, Hawthorne's Twice Told Tales, Longfellow's Evangeline, George Eliot's Silas Marner and a special knowledge of the subject matter, form and construction of the following works: Shakespeare's Merchant of Venice, Burke's Speech on Conciliation with America, Scott's Marmion, Macaulay's Life of Samuel Johnston.

History.—Of the United States (Fiske, Montgomery or Thomas); of Greece (Oman's History, with Mahaffy's Old Greek Life, or Myer's History); Roman (Allen's, pages 1-224 and Preston and Dodge's private life of the Romans). The history of England (Gardner is suggested) will be accepted as an equivalent of Greek and Roman history together.

Algebra.—To the end of Quadratic equations.

Plane geometry (as in the first five books of Chauvenet's or Wentworth's geometry).

In addition to the above, the applicant has his choice of being examined in any two of the following languages: Greek, Latin, French or German; or in any one of these languages and additional mathematics; or in any one of these languages and physics.

The most important changes from the present requirements in the college are in Greek, Roman, English and American history; algebra and plane geometry; Greek, Latin, French and German languages.

The members of the faculty of the law school are of the opinion that the best training for the study of law is a full college course of four years, as the student's future depends in a large measure on his standing in the professional school. A large number of those who study law are college graduates, and those who are not, it is claimed, cannot hope, except in rare instances, to compete successfully with the college men. A college course permits a wide latitude in the choice of subjects of study, but there is no one course of study absolutely necessary to those who desire to study law. The main thing to be obtained from a college course is culture and mental training, but they recommend to the student a thorough knowledge of history, of constitutional and administrative law, of politics and economics and of some one language other than English—preferably Latin—as being of great assistance in the study of law.

In order that the students may be influenced in taking a college course prior to entering the law school, all candidates for admission to the first-year class, possessing the degree of bachelor of arts or bachelor of science, or an equivalent degree from any recognised college or university, will be admitted without further examination. A diploma from an approved public high school will be accepted during 1897, in lieu of an examination in those subjects covered by the diploma.

The post-graduate course in law, which extends through two years, and aims not only to broaden and deepen the legal knowledge acquired in the law school course, but also to enable students to pursue advanced work with a view to special investigation and research, is suspended during the current year, pending certain modifications in the system.

That the new system may not be a too radical departure for the medical school, the requirements in that department will be gradually increased each year, beginning with 1897. At that time candidates for admission who do not possess college degrees will be required to pass examinations in English grammar, United States history, geography, arithmetic and algebra, physics and Latin grammar or plane geometry. The requirements in these studies will be increased for 1898. By 1899 those entering the first-year class will be required to pass examinations in the subjects at present required for entrance to the freshman class in the college of the university.

In addition to the requirements for 1899 the faculty of the medical school recommends that young men, before entering upon the study of medicine in that department, should have previously taken the degree either of bachelor of arts or bachelor of science at some college or university where the standing is equivalent to that of the University of Pennsylvania. Should this be impracticable two years' time should be devoted to the study of chemistry or biology, with laboratory work in each, amounting to six hours a week, for eight months in each year, mammalian and human anatomy, histology and physiology, including the practical use of the microscope and culture methods in bacteriology. Free hand drawing is also an important preliminary study.

Any candidate who may have received a degree in arts or science from a college recognised by the university will be admitted without examination. Students who have satisfactorily pursued the two-year course in biology at the university will be exempt from the entrance examination; and, until 1899, any candidate who has passed the entrance examination of a recognised college, or is a graduate of a recognised normal or high school or academy of equal rank, may enter without examination.

After this new plan has been thoroughly established in the medical and law schools, it is more than probable that the next step taken in the interest of higher education will be in requiring from all students entering these two departments a degree from some college of the same standing in the educational world as the College of the University of Pennsylvania.

---

### THE NEW IOWA MEDICAL PRACTICE LAW,

It is believed, (*Va. Med. Semi-Monthly*,) will compel quackery of every kind to look elsewhere than in Iowa for its victims. Christian science, osteopathy, the magnetic fraud, the traveling quack—all will be required to move out of the state, or show good cause for tarrying. The only compromise in the law is an amendment providing that those who have practised five consecutive years—three of which shall be in one locality in Iowa—may continue their practice. This lets in a few frauds, but the doors are tightly closed behind them. The backbone of the law provides that a diploma from a school recognised as in good standing, admits only to examination of the candidate. The law will practically enforce itself. It provides a penalty of not less than $300, and commitment until paid. Few will care to risk the consequences of violation. The district court only has jurisdiction. The medical student clause permits only those to practise under a preceptor who has had two courses of lectures in a recognised medical college.

---

### IDAHO STATE BOARD OF MEDICAL EXAMINERS.

This new organisation is getting in shape for business (*Med. Sentinel*) and registering the physicians under the new law. Dr. C. L. Sweet, the secretary, has a large amount of work to do, and in fact the full board, in reörganising the state on a medical basis, is kept busy. The physicians of the state have been furnished with blanks and are given the privilege of having their diplomas registered where they already have certificates. The first examination session will be held on September 6th, at Boise City, in the state house, at 10 A. M., and everything will be running along smoothly and nicely in a short time. The *Sentinel* predicts that scarcely twelve months will have elapsed until Idaho will be known all over the country as having in operation one of the best medical laws in existence and that this law is in the hands of a board competent and eager to enforce the same.

---

### THE WISCONSIN EXAMINING BOARD.

The State Board of Medical Examiners, (*Peoria Med. Jour.*, August, 1897,) under the new Wisconsin law, was organised a few weeks ago by the election of Dr. Samuel Bell, of Beloit, as president and Dr. H. M. Ludwig, of Richland Centre, as secretary and treasurer. The former is a member of the regular profession and the latter an "eclectic."

It was decided that all examinations shall be conducted in the English language and that the examination fee be $10 and the license fee $5. Rules were also adopted for the government of examinations, the form of application for examination and registration now used by the Iowa State Board being selected, and the Minnesota form of certificate decided upon.

